# Supplementary material for: Effect of Healthy Transitions intervention in improving family planning uptake among adolescents and young women in Western Nepal: A pre-and post-intervention study
Source: PLoS One. 2023 Jun 9;18(6):e0286705. doi: 10.1371/journal.pone.0286705 (PMC10256217; doi:10.1371/journal.pone.0286705)
Supplement: S1 Data — (PDF) [file pone.0286705.s001.pdf]

```
  ___  ____  ____  ____  ____(R)
 /__    /   ____/   /   ____/
___/   /   /___/   /   /___/
  Statistics/Data Analysis
```

```
  ___  ____  ____  ____  ____ (R)
 /__    /   ____/   /   ____/
___/   /   /___/   /   /___/   15.1   Copyright 1985-2017 StataCorp LLC
  Statistics/Data Analysis            StataCorp
                                      4905 Lakeway Drive
     Special Edition                  College Station, Texas 77845 USA
                                      800-STATA-PC        http://www.stata.com
                                      979-696-4600        stata@stata.com
                                      979-696-4601 (fax)
```

Single-user Stata perpetual license:
       Serial number: 401506250534
         Licensed to: Tara
                       Research

Notes:
1. Unicode is supported; see help unicode advice.
2. Maximum number of variables is set to 5000; see help set maxvar.
3. New update available; type -update all-

Checking for updates...
(contacting http://www.stata.com)

Update status

Last check for updates:	**04 Apr 2023**	
New update available:	**03 Feb 2020**	(what's new)
Current update level:	**21 Nov 2017**	(what's new)

Comment

Stata 17 available

Find out more: stata.com/new-in-stata

New features:

Tables	PyStata
Bayesian econometrics	Jupyter Notebook with Stata
Interval-censored Cox model	Faster Stata
Differences-in-differences (DID)	Bayesian mutilevel modeling
Bayesian VAR	New functions for dates and times
Multivariate meta-analysis	Leave-one-out meta-analysis
Treatment-effects lasso	Galbraith plots
Panel-data multinomial logit	Bayesian panel-data models
Zero-inflated ordered logit	Nonparametric tests for trend
Bayesian IRF and FEVD analysis	Lasso with clustered data
Bayesian dynamic forecasting	BIC for lasso penalty selection
Do-file Editor enhancements	Bayesian linear and nonlinear DSGEs
Intel Math Kernel Library (MKL)	H2O integration
Stata on Apple Silicon	Java integration
JDBC	

and more

Possible actions

Install available updates (or type -update all-)

Click to edit automatic update checking preferences

```
1 . use "C:\Users\r.bhandari\Downloads\HTNYP_public_use.dta"

2 . mcc round S316

                 | Controls               |
Cases            |   Exposed   Unexposed  |      Total
-----------------+------------------------+-----------
         Exposed |       329           0  |        329
       Unexposed |         0           0  |          0
-----------------+------------------------+-----------
           Total |       329           0  |        329

McNemar's chi2(1) =          .    Prob > chi2 =      .
Exact McNemar significance probability       = 1.0000

Proportion with factor
        Cases               1
        Controls            1     [95% Conf. Interval]
                   ---------     --------------------
        difference          0     -.0030395   .0030395
        ratio               1             1          1
        rel. diff.          .             .          .

        odds ratio          .             .          .   (exact)

3 . mcc S316 round

                 | Controls               |
Cases            |   Exposed   Unexposed  |      Total
-----------------+------------------------+-----------
         Exposed |       329           0  |        329
       Unexposed |         0           0  |          0
-----------------+------------------------+-----------
           Total |       329           0  |        329

McNemar's chi2(1) =          .    Prob > chi2 =      .
Exact McNemar significance probability       = 1.0000

Proportion with factor
        Cases               1
        Controls            1     [95% Conf. Interval]
                   ---------     --------------------
        difference          0     -.0030395   .0030395
        ratio               1             1          1
        rel. diff.          .             .          .

        odds ratio          .             .          .   (exact)

4 . tab round

      round |      Freq.     Percent        Cum.
------------+-----------------------------------
          1 |        786       50.00       50.00
          2 |        786       50.00      100.00
------------+-----------------------------------
      Total |      1,572      100.00
```

5 . tab S316

S316. Are you currently doing something or using any method to delay or avoid ge	Freq.	Percent	Cum.
Yes	**168**	**51.06**	**51.06**
No	**161**	**48.94**	**100.00**
Total	**329**	**100.00**	

6 . tab round S316

round	S316. Are you currently doing something or using any method to delay or avoid ge: Yes	No	Total
1	**87**	**77**	**164**
2	**81**	**84**	**165**
Total	**168**	**161**	**329**

7 . mcc round S316

Cases	Controls Exposed	Unexposed	Total
Exposed	**329**	**0**	**329**
Unexposed	**0**	**0**	**0**
Total	**329**	**0**	**329**

```
McNemar's chi2(1) =          .    Prob > chi2 =      .
Exact McNemar significance probability       = 1.0000

Proportion with factor
        Cases                 1
        Controls              1     [95% Conf. Interval]
                      ---------     --------------------
        difference            0     -.0030395   .0030395
        ratio                 1             1          1
        rel. diff.            .             .          .

        odds ratio            .             .          .   (exact)
```

```
 8 . tab b_evmar

   Baseline: |
        Ever |
     married |      Freq.     Percent        Cum.
-------------+-----------------------------------
          No |        992       63.10       63.10
         Yes |        580       36.90      100.00
-------------+-----------------------------------
       Total |      1,572      100.00

 9 . tab round b_evmar

           |    Baseline: Ever
           |       married
     round |        No        Yes |     Total
-----------+----------------------+----------
         1 |       496        290 |       786
         2 |       496        290 |       786
-----------+----------------------+----------
     Total |       992        580 |     1,572


10 . tab round S316

           |     S316. Are you
           |    currently doing
           |  something or using
           |  any method to delay
           |      or avoid ge
     round |       Yes         No |     Total
-----------+----------------------+----------
         1 |        87         77 |       164
         2 |        81         84 |       165
-----------+----------------------+----------
     Total |       168        161 |       329


11 . mcci 87 77 81 84

                 | Controls               |
Cases            |   Exposed   Unexposed  |      Total
-----------------+------------------------+-----------
         Exposed |        87          77  |        164
       Unexposed |        81          84  |        165
-----------------+------------------------+-----------
           Total |       168         161  |        329

McNemar's chi2(1) =      0.10    Prob > chi2 = 0.7503
Exact McNemar significance probability       = 0.8115

Proportion with factor
        Cases       .4984802
        Controls    .5106383     [95% Conf. Interval]
                   ---------     --------------------
        difference -.0121581     -.0900686   .0657525
        ratio       .9761905      .8415413   1.132384
        rel. diff. -.0248447     -.1797548   .1300654

        odds ratio  .9506173      .6867165   1.315003   (exact)
```

```
12 . mcc round S316 if b_evmar ==1

                 | Controls               |
Cases            |   Exposed   Unexposed  |      Total
-----------------+------------------------+-----------
         Exposed |       323           0  |        323
       Unexposed |         0           0  |          0
-----------------+------------------------+-----------
           Total |       323           0  |        323

McNemar's chi2(1) =          .    Prob > chi2 =      .
Exact McNemar significance probability       = 1.0000

Proportion with factor
        Cases             1
        Controls          1     [95% Conf. Interval]
                  ---------     --------------------
        difference        0     -.003096    .003096
        ratio             1            1          1
        rel. diff.        .            .          .

        odds ratio        .            .          .   (exact)

13 . mcc round S316

                 | Controls               |
Cases            |   Exposed   Unexposed  |      Total
-----------------+------------------------+-----------
         Exposed |       329           0  |        329
       Unexposed |         0           0  |          0
-----------------+------------------------+-----------
           Total |       329           0  |        329

McNemar's chi2(1) =          .    Prob > chi2 =      .
Exact McNemar significance probability       = 1.0000

Proportion with factor
        Cases             1
        Controls          1     [95% Conf. Interval]
                  ---------     --------------------
        difference        0    -.0030395   .0030395
        ratio             1            1          1
        rel. diff.        .            .          .

        odds ratio        .            .          .   (exact)

14 . mcci 29 71 38 62

                 | Controls               |
Cases            |   Exposed   Unexposed  |      Total
-----------------+------------------------+-----------
         Exposed |        29          71  |        100
       Unexposed |        38          62  |        100
-----------------+------------------------+-----------
           Total |        67         133  |        200

McNemar's chi2(1) =       9.99    Prob > chi2 = 0.0016
Exact McNemar significance probability       = 0.0020

Proportion with factor
        Cases            .5
        Controls       .335     [95% Conf. Interval]
                  ---------     --------------------
        difference     .165     .0602751   .2697249
        ratio      1.492537       1.1624   1.916438
        rel. diff. .2481203     .1147117   .3815289
```

```
        odds ratio  1.868421      1.242935   2.848266   (exact)

15 . mcci 26 74 33 67

                 | Controls
Cases            |  Exposed   Unexposed  |      Total
-----------------+-----------------------+-----------
         Exposed |       26          74  |        100
       Unexposed |       33          67  |        100
-----------------+-----------------------+-----------
           Total |       59         141  |        200

McNemar's chi2(1) =     15.71    Prob > chi2 = 0.0001
Exact McNemar significance probability       = 0.0001

Proportion with factor
        Cases             .5
        Controls        .295     [95% Conf. Interval]
                   ---------     --------------------
        difference      .205      .1026927   .3073073
        ratio       1.694915      1.301721   2.206876
        rel. diff.  .2907801      .1696893    .411871

        odds ratio  2.242424       1.46892   3.490135   (exact)

16 . mcci 92 8 99 1

                 | Controls
Cases            |  Exposed   Unexposed  |      Total
-----------------+-----------------------+-----------
         Exposed |       92           8  |        100
       Unexposed |       99           1  |        100
-----------------+-----------------------+-----------
           Total |      191           9  |        200

McNemar's chi2(1) =     77.39    Prob > chi2 = 0.0000
Exact McNemar significance probability       = 0.0000

Proportion with factor
        Cases             .5
        Controls        .955     [95% Conf. Interval]
                   ---------     --------------------
        difference     -.455     -.5393695  -.3706305
        ratio       .5235602       .452123   .6062847
        rel. diff. -10.11111     -17.62001  -2.602213

        odds ratio  .0808081      .0339451   .1655169   (exact)

17 . mcci 92 7 99 1

                 | Controls
Cases            |  Exposed   Unexposed  |      Total
-----------------+-----------------------+-----------
         Exposed |       92           7  |         99
       Unexposed |       99           1  |        100
-----------------+-----------------------+-----------
           Total |      191           8  |        199

McNemar's chi2(1) =     79.85    Prob > chi2 = 0.0000
Exact McNemar significance probability       = 0.0000
```

```
Proportion with factor
        Cases       .4974874
        Controls     .959799     [95% Conf. Interval]
                   ---------     --------------------
        difference -.4623116     -.5458006  -.3788225
        ratio       .5183246      .4475801    .600251
        rel. diff.     -11.5     -20.41797  -2.582029

        odds ratio  .0707071      .0277078   .1511951   (exact)

18 . mcci 39 61 63 37

                 | Controls              |
Cases            |   Exposed   Unexposed |      Total
-----------------+-----------------------+-----------
         Exposed |        39          61 |        100
       Unexposed |        63          37 |        100
-----------------+-----------------------+-----------
           Total |       102          98 |        200

McNemar's chi2(1) =      0.03    Prob > chi2 = 0.8575
Exact McNemar significance probability       = 0.9285

Proportion with factor
        Cases             .5
        Controls         .51     [95% Conf. Interval]
                   ---------     --------------------
        difference      -.01     -.1241174   .1041174
        ratio       .9803922       .789856   1.216891
        rel. diff. -.0204082     -.2453757   .2045594

        odds ratio   .968254      .6695969   1.399301   (exact)

19 . mcci 67 37 33 63

                 | Controls              |
Cases            |   Exposed   Unexposed |      Total
-----------------+-----------------------+-----------
         Exposed |        67          37 |        104
       Unexposed |        33          63 |         96
-----------------+-----------------------+-----------
           Total |       100         100 |        200

McNemar's chi2(1) =      0.23    Prob > chi2 = 0.6326
Exact McNemar significance probability       = 0.7202

Proportion with factor
        Cases            .52
        Controls          .5     [95% Conf. Interval]
                   ---------     --------------------
        difference       .02     -.0669443   .1069443
        ratio           1.04      .8855227   1.221426
        rel. diff.       .04     -.1206692   .2006692

        odds ratio  1.121212      .6821639    1.84999   (exact)
```

20 . mcci 67 33 37 63

Cases	Controls Exposed	Unexposed	Total
Exposed	67	33	100
Unexposed	37	63	100
Total	104	96	200

McNemar's chi2(1) = 0.23   Prob > chi2 = 0.6326
Exact McNemar significance probability = 0.7202

Proportion with factor		[95% Conf.	Interval]	
Cases	.5			
Controls	.52			
difference	-.02	-.1069443	.0669443	
ratio	.9615385	.8187155	1.129277	
rel. diff.	-.0416667	-.2160039	.1326706	
odds ratio	.8918919	.5405435	1.465923	(exact)

21 . mcci 27 38 73 62

Cases	Controls Exposed	Unexposed	Total
Exposed	27	38	65
Unexposed	73	62	135
Total	100	100	200

McNemar's chi2(1) = 11.04   Prob > chi2 = 0.0009
Exact McNemar significance probability = 0.0012

Proportion with factor		[95% Conf.	Interval]	
Cases	.325			
Controls	.5			
difference	-.175	-.2803585	-.0696415	
ratio	.65	.5031291	.8397447	
rel. diff.	-.35	-.5899255	-.1100745	
odds ratio	.5205479	.3421121	.780705	(exact)

22 . mcci 31 38 69 62

Cases	Controls Exposed	Unexposed	Total
Exposed	31	38	69
Unexposed	69	62	131
Total	100	100	200

McNemar's chi2(1) = 8.98   Prob > chi2 = 0.0027
Exact McNemar significance probability = 0.0035

Proportion with factor		[95% Conf.	Interval]
Cases	.345		
Controls	.5		
difference	-.155	-.2590679	-.0509321
ratio	.69	.5405683	.8807397
rel. diff.	-.31	-.5420468	-.0779532

```
        odds ratio  .5507246       .360556   .8298786   (exact)

23 . mcci 24 31 76 69

                 |   Controls             |
Cases            |    Exposed   Unexposed |      Total
-----------------+------------------------+-----------
         Exposed |         24          31 |         55
       Unexposed |         76          69 |        145
-----------------+------------------------+-----------
           Total |        100         100 |        200

McNemar's chi2(1) =     18.93    Prob > chi2 = 0.0000
Exact McNemar significance probability       = 0.0000

Proportion with factor
        Cases           .275
        Controls          .5     [95% Conf. Interval]
                   ---------     --------------------
        difference     -.225     -.3264548  -.1235452
        ratio            .55      .4184441   .7229161
        rel. diff.      -.45     -.6941316  -.2058684

        odds ratio  .4078947      .2595654   .6269852   (exact)

24 . mcci 38 46 62 74

                 |   Controls             |
Cases            |    Exposed   Unexposed |      Total
-----------------+------------------------+-----------
         Exposed |         38          46 |         84
       Unexposed |         62          74 |        136
-----------------+------------------------+-----------
           Total |        100         120 |        220

McNemar's chi2(1) =      2.37    Prob > chi2 = 0.1237
Exact McNemar significance probability       = 0.1486

Proportion with factor
        Cases       .3818182
        Controls    .4545455     [95% Conf. Interval]
                   ---------     --------------------
        difference -.0727273     -.1693569   .0239023
        ratio            .84      .6726081   1.049051
        rel. diff. -.1333333     -.3140331   .0473664

        odds ratio  .7419355      .4953696   1.104154   (exact)

25 . mcci 29 36 71 64

                 |   Controls             |
Cases            |    Exposed   Unexposed |      Total
-----------------+------------------------+-----------
         Exposed |         29          36 |         65
       Unexposed |         71          64 |        135
-----------------+------------------------+-----------
           Total |        100         100 |        200

McNemar's chi2(1) =     11.45    Prob > chi2 = 0.0007
Exact McNemar significance probability       = 0.0009
```

```
Proportion with factor
        Cases           .325
        Controls          .5       [95% Conf. Interval]
                   ---------       --------------------
        difference     -.175       -.278426   -.071574
        ratio            .65       .5054778   .8358429
        rel. diff.      -.35      -.5855629  -.1144371

        odds ratio  .5070423       .3297364   .7675278   (exact)
```

26 . mcci 35 50 65 50

Cases	Controls Exposed	Unexposed	Total
Exposed	35	50	85
Unexposed	65	50	115
Total	100	100	200

```
McNemar's chi2(1) =      1.96    Prob > chi2 = 0.1619
Exact McNemar significance probability       = 0.1915

Proportion with factor
        Cases           .425
        Controls          .5       [95% Conf. Interval]
                   ---------       --------------------
        difference     -.075      -.1845761   .0345761
        ratio            .85       .6767224   1.067646
        rel. diff.      -.15      -.3753959   .0753959

        odds ratio  .7692308       .5211412   1.129508   (exact)
```

27 . mcci 26 43 74 67

Cases	Controls Exposed	Unexposed	Total
Exposed	26	43	69
Unexposed	74	67	141
Total	100	110	210

```
McNemar's chi2(1) =      8.21    Prob > chi2 = 0.0042
Exact McNemar significance probability       = 0.0053

Proportion with factor
        Cases       .3285714
        Controls    .4761905       [95% Conf. Interval]
                   ---------       --------------------
        difference -.147619       -.2513406  -.0438975
        ratio            .69       .5345742   .8906154
        rel. diff. -.2818182      -.5000216  -.0636148

        odds ratio  .5810811       .3894539   .8574592   (exact)
```

28 . mcci 26 43 74 75

Cases	Controls Exposed	Unexposed	Total
Exposed	26	43	69
Unexposed	74	75	149
Total	100	118	218

McNemar's chi2(1) = 8.21   Prob > chi2 = 0.0042
Exact McNemar significance probability = 0.0053

Proportion with factor		[95% Conf. Interval]		
Cases	.3165138			
Controls	.4587156			
difference	-.1422018	-.2421882	-.0422154	
ratio	.69	.5345742	.8906154	
rel. diff.	-.2627119	-.4646002	-.0608236	
odds ratio	.5810811	.3894539	.8574592	(exact)

29 . mcci 32 36 68 64

Cases	Controls Exposed	Unexposed	Total
Exposed	32	36	68
Unexposed	68	64	132
Total	100	100	200

McNemar's chi2(1) = 9.85   Prob > chi2 = 0.0017
Exact McNemar significance probability = 0.0022

Proportion with factor		[95% Conf. Interval]		
Cases	.34			
Controls	.5			
difference	-.16	-.2624479	-.0575521	
ratio	.68	.5336314	.8665158	
rel. diff.	-.32	-.5496422	-.0903578	
odds ratio	.5294118	.3432716	.8043097	(exact)

30 . mcci 35 32 65 68

Cases	Controls Exposed	Unexposed	Total
Exposed	35	32	67
Unexposed	65	68	133
Total	100	100	200

McNemar's chi2(1) = 11.23   Prob > chi2 = 0.0008
Exact McNemar significance probability = 0.0010

Proportion with factor		[95% Conf. Interval]	
Cases	.335		
Controls	.5		
difference	-.165	-.263769	-.066231
ratio	.67	.5292437	.8481914
rel. diff.	-.33	-.5526177	-.1073823

```
        odds ratio  .4923077      .3119173   .7628627   (exact)

31 . mcci 34 33 66 67

                 | Controls
Cases            |   Exposed   Unexposed  |       Total
-----------------+------------------------+------------
         Exposed |        34          33  |          67
       Unexposed |        66          67  |         133
-----------------+------------------------+------------
           Total |       100         100  |         200

McNemar's chi2(1) =     11.00    Prob > chi2 = 0.0009
Exact McNemar significance probability       = 0.0012

Proportion with factor
        Cases            .335
        Controls           .5     [95% Conf. Interval]
                    ---------     --------------------
        difference      -.165     -.2647876  -.0652124
        ratio             .67      .5279651   .8502455
        rel. diff.       -.33     -.5549011  -.1050989

        odds ratio         .5      .3188376   .7704782   (exact)

32 . mcci 25 45 75 55

                 | Controls
Cases            |   Exposed   Unexposed  |       Total
-----------------+------------------------+------------
         Exposed |        25          45  |          70
       Unexposed |        75          55  |         130
-----------------+------------------------+------------
           Total |       100         100  |         200

McNemar's chi2(1) =      7.50    Prob > chi2 = 0.0062
Exact McNemar significance probability       = 0.0078

Proportion with factor
        Cases             .35
        Controls           .5     [95% Conf. Interval]
                    ---------     --------------------
        difference       -.15     -.2603196  -.0396804
        ratio              .7      .5415638   .9047873
        rel. diff.        -.3     -.5447994  -.0552006

        odds ratio         .6      .4051134   .8797412   (exact)

33 . mcci 25 42 75 58

                 | Controls
Cases            |   Exposed   Unexposed  |       Total
-----------------+------------------------+------------
         Exposed |        25          42  |          67
       Unexposed |        75          58  |         133
-----------------+------------------------+------------
           Total |       100         100  |         200

McNemar's chi2(1) =      9.31    Prob > chi2 = 0.0023
Exact McNemar significance probability       = 0.0029
```

```
Proportion with factor
        Cases              .335
        Controls             .5     [95% Conf. Interval]
                     ---------     ---------------------
        difference       -.165     -.2735053  -.0564947
        ratio              .67      .5171202   .8680766
        rel. diff.        -.33     -.5744932  -.0855068

        odds ratio         .56      .3743809   .8277368   (exact)

34 . mcci 33 38 67 62

                 | Controls                |
Cases            |   Exposed   Unexposed   |      Total
-----------------+-------------------------+-----------
         Exposed |        33          38   |         71
       Unexposed |        67          62   |        129
-----------------+-------------------------+-----------
           Total |       100         100   |        200

McNemar's chi2(1) =      8.01    Prob > chi2 = 0.0047
Exact McNemar significance probability       = 0.0060

Proportion with factor
        Cases              .355
        Controls             .5     [95% Conf. Interval]
                     ---------     ---------------------
        difference       -.145      -.248387   -.041613
        ratio              .71      .5594286   .9010981
        rel. diff.        -.29     -.5181065  -.0618935

        odds ratio    .5671642      .3705481   .8568428   (exact)

35 . mcci 27 36 73 64

                 | Controls                |
Cases            |   Exposed   Unexposed   |      Total
-----------------+-------------------------+-----------
         Exposed |        27          36   |         63
       Unexposed |        73          64   |        137
-----------------+-------------------------+-----------
           Total |       100         100   |        200

McNemar's chi2(1) =     12.56    Prob > chi2 = 0.0004
Exact McNemar significance probability       = 0.0005

Proportion with factor
        Cases              .315
        Controls             .5     [95% Conf. Interval]
                     ---------     ---------------------
        difference       -.185     -.2890485  -.0809515
        ratio              .63        .48683   .8152743
        rel. diff.        -.37     -.6095089  -.1304911

        odds ratio    .4931507      .3212931   .7448074   (exact)
```

36 . mcci 38 30 62 70

Cases	Controls Exposed	Unexposed	Total
Exposed	38	30	68
Unexposed	62	70	132
Total	100	100	200

McNemar's chi2(1) = 11.13   Prob > chi2 = 0.0008
Exact McNemar significance probability = 0.0011

Proportion with factor			
Cases	.34		
Controls	.5	[95% Conf.	Interval]
difference	-.16	-.2563436	-.0636564
ratio	.68	.5413779	.8541168
rel. diff.	-.32	-.5359877	-.1040123
odds ratio	.483871	.302022	.7598215 (exact)

37 . mcci 23 30 77 70

Cases	Controls Exposed	Unexposed	Total
Exposed	23	30	53
Unexposed	77	70	147
Total	100	100	200

McNemar's chi2(1) = 20.64   Prob > chi2 = 0.0000
Exact McNemar significance probability = 0.0000

Proportion with factor			
Cases	.265		
Controls	.5	[95% Conf.	Interval]
difference	-.235	-.3359957	-.1340043
ratio	.53	.4011726	.7001973
rel. diff.	-.47	-.7158095	-.2241905
odds ratio	.3896104	.2466073	.6011999 (exact)

38 . mcci 32 57 68 43

Cases	Controls Exposed	Unexposed	Total
Exposed	32	57	89
Unexposed	68	43	111
Total	100	100	200

McNemar's chi2(1) = 0.97   Prob > chi2 = 0.3252
Exact McNemar significance probability = 0.3712

Proportion with factor			
Cases	.445		
Controls	.5	[95% Conf.	Interval]
difference	-.055	-.1692998	.0592998
ratio	.89	.7055259	1.122709
rel. diff.	-.11	-.3408684	.1208684

```
        odds ratio  .8382353      .5789865   1.209703   (exact)

39 . mcci 27 38 77 62

                 | Controls               |
Cases            |  Exposed   Unexposed   |      Total
-----------------+------------------------+-----------
         Exposed |       27          38   |         65
       Unexposed |       77          62   |        139
-----------------+------------------------+-----------
           Total |      104         100   |        204

McNemar's chi2(1) =     13.23    Prob > chi2 = 0.0003
Exact McNemar significance probability       = 0.0004

Proportion with factor
        Cases       .3186275
        Controls    .5098039     [95% Conf. Interval]
                   ---------     --------------------
        difference -.1911765     -.2957133  -.0866397
        ratio           .625      .4840144   .8070524
        rel. diff.      -.39     -.6378018  -.1421982

        odds ratio  .4935065      .3254703   .7369959   (exact)

40 . mcci 27 38 73 62

                 | Controls               |
Cases            |  Exposed   Unexposed   |      Total
-----------------+------------------------+-----------
         Exposed |       27          38   |         65
       Unexposed |       73          62   |        135
-----------------+------------------------+-----------
           Total |      100         100   |        200

McNemar's chi2(1) =     11.04    Prob > chi2 = 0.0009
Exact McNemar significance probability       = 0.0012

Proportion with factor
        Cases           .325
        Controls          .5     [95% Conf. Interval]
                   ---------     --------------------
        difference     -.175     -.2803585  -.0696415
        ratio            .65      .5031291   .8397447
        rel. diff.      -.35     -.5899255  -.1100745

        odds ratio  .5205479      .3421121    .780705   (exact)

41 . mcci 38 62 27 73

                 | Controls               |
Cases            |  Exposed   Unexposed   |      Total
-----------------+------------------------+-----------
         Exposed |       38          62   |        100
       Unexposed |       27          73   |        100
-----------------+------------------------+-----------
           Total |       65         135   |        200

McNemar's chi2(1) =     13.76    Prob > chi2 = 0.0002
Exact McNemar significance probability       = 0.0003
```

```
Proportion with factor
        Cases              .5
        Controls         .325     [95% Conf. Interval]
                    ---------     --------------------
        difference       .175      .0807866   .2692134
        ratio        1.538462      1.223162   1.935037
        rel. diff.   .2592593      .1413786   .3771399

        odds ratio   2.296296      1.439525   3.753994   (exact)

42 . mcci 31 69 38 62

                 | Controls               |
Cases            |   Exposed   Unexposed  |      Total
-----------------+------------------------+-----------
         Exposed |        31          69  |        100
       Unexposed |        38          62  |        100
-----------------+------------------------+-----------
           Total |        69         131  |        200

McNemar's chi2(1) =      8.98    Prob > chi2 = 0.0027
Exact McNemar significance probability       = 0.0035

Proportion with factor
        Cases              .5
        Controls         .345     [95% Conf. Interval]
                    ---------     --------------------
        difference       .155      .0509321   .2590679
        ratio        1.449275      1.135409   1.849905
        rel. diff.   .2366412      .1014237   .3718588

        odds ratio   1.815789      1.204996   2.773495   (exact)

43 . mcci 27 73 36 64

                 | Controls               |
Cases            |   Exposed   Unexposed  |      Total
-----------------+------------------------+-----------
         Exposed |        27          73  |        100
       Unexposed |        36          64  |        100
-----------------+------------------------+-----------
           Total |        63         137  |        200

McNemar's chi2(1) =     12.56    Prob > chi2 = 0.0004
Exact McNemar significance probability       = 0.0005

Proportion with factor
        Cases              .5
        Controls         .315     [95% Conf. Interval]
                    ---------     --------------------
        difference       .185      .0809515   .2890485
        ratio        1.587302      1.226581   2.054105
        rel. diff.    .270073      .1424642   .3976818

        odds ratio   2.027778      1.342629   3.112423   (exact)
```

```
44 . mcci 38 62 30 70

                 | Controls               |
Cases            |   Exposed   Unexposed  |      Total
-----------------+------------------------+-----------
         Exposed |        38          62  |        100
       Unexposed |        30          70  |        100
-----------------+------------------------+-----------
           Total |        68         132  |        200

McNemar's chi2(1) =     11.13    Prob > chi2 = 0.0008
Exact McNemar significance probability       = 0.0011

Proportion with factor
        Cases             .5
        Controls         .34     [95% Conf. Interval]
                   ---------     --------------------
        difference       .16      .0636564   .2563436
        ratio       1.470588        1.1708   1.847138
        rel. diff.  .2424242      .1184644   .3663841

        odds ratio  2.066667      1.316099   3.311017   (exact)

45 . mcci 23 77 30 70

                 | Controls               |
Cases            |   Exposed   Unexposed  |      Total
-----------------+------------------------+-----------
         Exposed |        23          77  |        100
       Unexposed |        30          70  |        100
-----------------+------------------------+-----------
           Total |        53         147  |        200

McNemar's chi2(1) =     20.64    Prob > chi2 = 0.0000
Exact McNemar significance probability       = 0.0000

Proportion with factor
        Cases             .5
        Controls        .265     [95% Conf. Interval]
                   ---------     --------------------
        difference      .235      .1340043   .3359957
        ratio       1.886792      1.428169   2.492692
        rel. diff.  .3197279      .2059746   .4334812

        odds ratio  2.566667       1.66334   4.055031   (exact)

46 . mcci 32 68 57 43

                 | Controls               |
Cases            |   Exposed   Unexposed  |      Total
-----------------+------------------------+-----------
         Exposed |        32          68  |        100
       Unexposed |        57          43  |        100
-----------------+------------------------+-----------
           Total |        89         111  |        200

McNemar's chi2(1) =      0.97    Prob > chi2 = 0.3252
Exact McNemar significance probability       = 0.3712

Proportion with factor
        Cases             .5
        Controls        .445     [95% Conf. Interval]
                   ---------     --------------------
        difference      .055     -.0592998   .1692998
        ratio       1.123596       .890703   1.417382
        rel. diff.  .0990991     -.0882789   .2864771
```

```
        odds ratio  1.192982      .8266492   1.727156   (exact)

47 . mcci 11 89 17 83

                 | Controls               |
Cases            |   Exposed   Unexposed  |       Total
-----------------+------------------------+------------
         Exposed |        11          89  |         100
       Unexposed |        17          83  |         100
-----------------+------------------------+------------
           Total |        28         172  |         200

McNemar's chi2(1) =     48.91    Prob > chi2 = 0.0000
Exact McNemar significance probability       = 0.0000

Proportion with factor
        Cases             .5
        Controls         .14     [95% Conf. Interval]
                   ---------     --------------------
        difference       .36      .2673039   .4526961
        ratio       3.571429      2.439071   5.229492
        rel. diff.  .4186047      .3291489   .5080604

        odds ratio  5.235294      3.093052   9.385099   (exact)

48 . mcci 30 70 36 64

                 | Controls               |
Cases            |   Exposed   Unexposed  |       Total
-----------------+------------------------+------------
         Exposed |        30          70  |         100
       Unexposed |        36          64  |         100
-----------------+------------------------+------------
           Total |        66         134  |         200

McNemar's chi2(1) =     10.91    Prob > chi2 = 0.0010
Exact McNemar significance probability       = 0.0012

Proportion with factor
        Cases             .5
        Controls         .33     [95% Conf. Interval]
                   ---------     --------------------
        difference       .17      .0668941   .2731059
        ratio       1.515152      1.181906   1.942358
        rel. diff.  .2537313      .1236414   .3838213

        odds ratio  1.944444      1.283019    2.99287   (exact)

49 . mcci 38 62 46 54

                 | Controls               |
Cases            |   Exposed   Unexposed  |       Total
-----------------+------------------------+------------
         Exposed |        38          62  |         100
       Unexposed |        46          54  |         100
-----------------+------------------------+------------
           Total |        84         116  |         200

McNemar's chi2(1) =      2.37    Prob > chi2 = 0.1237
Exact McNemar significance probability       = 0.1486
```

```
Proportion with factor
        Cases               .5
        Controls           .42      [95% Conf. Interval]
                  ---------     --------------------
        difference         .08     -.0262374   .1862374
        ratio         1.190476      .9532428    1.48675
        rel. diff.     .137931      -.025101   .3009631

        odds ratio    1.347826      .9056707   2.018695   (exact)

50 . mcci 29 71 36 64

                 | Controls               |
Cases            |   Exposed   Unexposed  |      Total
-----------------+------------------------+-----------
         Exposed |        29          71  |        100
       Unexposed |        36          64  |        100
-----------------+------------------------+-----------
           Total |        65         135  |        200

McNemar's chi2(1) =     11.45    Prob > chi2 = 0.0007
Exact McNemar significance probability       = 0.0009

Proportion with factor
        Cases               .5
        Controls          .325      [95% Conf. Interval]
                  ---------     --------------------
        difference        .175       .071574    .278426
        ratio         1.538462      1.196397   1.978326
        rel. diff.    .2592593      .1300066   .3885119

        odds ratio    1.972222      1.302884   3.032726   (exact)

51 . mcci 35 65 50 50

                 | Controls               |
Cases            |   Exposed   Unexposed  |      Total
-----------------+------------------------+-----------
         Exposed |        35          65  |        100
       Unexposed |        50          50  |        100
-----------------+------------------------+-----------
           Total |        85         115  |        200

McNemar's chi2(1) =      1.96    Prob > chi2 = 0.1619
Exact McNemar significance probability       = 0.1915

Proportion with factor
        Cases               .5
        Controls          .425      [95% Conf. Interval]
                  ---------     --------------------
        difference        .075     -.0345761   .1845761
        ratio         1.176471        .93664   1.477711
        rel. diff.    .1304348     -.0399969   .3008664

        odds ratio         1.3      .8853415   1.918866   (exact)
```

```
52 . mcci mcci 26 74 43 57
   'mcci' found where integer expected
   r(7);

53 . mcci 26 74 43 57

                 | Controls               |
Cases            |    Exposed   Unexposed |      Total
-----------------+------------------------+-----------
         Exposed |         26          74 |        100
       Unexposed |         43          57 |        100
-----------------+------------------------+-----------
           Total |         69         131 |        200

McNemar's chi2(1) =      8.21    Prob > chi2 = 0.0042
Exact McNemar significance probability       = 0.0053

Proportion with factor
        Cases              .5
        Controls         .345     [95% Conf. Interval]
                    ---------     --------------------
        difference       .155      .0461982   .2638018
        ratio        1.449275      1.122819   1.870648
        rel. diff.   .2366412      .0952462   .3780362

        odds ratio    1.72093      1.166236   2.567698   (exact)

54 . mcci 32 68 36 64

                 | Controls               |
Cases            |    Exposed   Unexposed |      Total
-----------------+------------------------+-----------
         Exposed |         32          68 |        100
       Unexposed |         36          64 |        100
-----------------+------------------------+-----------
           Total |         68         132 |        200

McNemar's chi2(1) =      9.85    Prob > chi2 = 0.0017
Exact McNemar significance probability       = 0.0022

Proportion with factor
        Cases              .5
        Controls          .34     [95% Conf. Interval]
                    ---------     --------------------
        difference        .16      .0575521   .2624479
        ratio        1.470588      1.154047   1.873953
        rel. diff.   .2424242      .1106277   .3742207

        odds ratio   1.888889      1.243302   2.913145   (exact)

55 . mcci 35 65 32 68

                 | Controls               |
Cases            |    Exposed   Unexposed |      Total
-----------------+------------------------+-----------
         Exposed |         35          65 |        100
       Unexposed |         32          68 |        100
-----------------+------------------------+-----------
           Total |         67         133 |        200

McNemar's chi2(1) =     11.23    Prob > chi2 = 0.0008
Exact McNemar significance probability       = 0.0010
```

```
Proportion with factor
        Cases               .5
        Controls          .335      [95% Conf. Interval]
                     ---------     --------------------
        difference        .165       .066231    .263769
        ratio         1.492537      1.178979   1.889489
        rel. diff.    .2481203      .1222694   .3739713

        odds ratio     2.03125      1.310852   3.205978   (exact)

56 . mcci 26 74 44 66

                 | Controls               |
Cases            |  Exposed   Unexposed   |      Total
-----------------+------------------------+-----------
         Exposed |       26          74   |        100
       Unexposed |       44          66   |        110
-----------------+------------------------+-----------
           Total |       70         140   |        210

McNemar's chi2(1) =      7.63    Prob > chi2 = 0.0057
Exact McNemar significance probability       = 0.0073

Proportion with factor
        Cases         .4761905
        Controls      .3333333      [95% Conf. Interval]
                     ---------     --------------------
        difference    .1428571      .0385693    .247145
        ratio         1.428571      1.107608   1.842544
        rel. diff.    .2142857      .0794846   .3490868

        odds ratio    1.681818      1.142787   2.501097   (exact)

57 . mcci 26 74 44 56

                 | Controls               |
Cases            |  Exposed   Unexposed   |      Total
-----------------+------------------------+-----------
         Exposed |       26          74   |        100
       Unexposed |       44          56   |        100
-----------------+------------------------+-----------
           Total |       70         130   |        200

McNemar's chi2(1) =      7.63    Prob > chi2 = 0.0057
Exact McNemar significance probability       = 0.0073

Proportion with factor
        Cases               .5
        Controls           .35      [95% Conf. Interval]
                     ---------     --------------------
        difference         .15      .0405963   .2594037
        ratio         1.428571      1.107608   1.842544
        rel. diff.    .2307692      .0871297   .3744088

        odds ratio    1.681818      1.142787   2.501097   (exact)
```

```
58 . mcci 21 79 50 50

                 │ Controls
Cases            │   Exposed   Unexposed  │       Total
─────────────────┼────────────────────────┼─────────────
         Exposed │        21          79  │         100
       Unexposed │        50          50  │         100
─────────────────┼────────────────────────┼─────────────
           Total │        71         129  │         200

McNemar's chi2(1) =      6.52    Prob > chi2 = 0.0107
Exact McNemar significance probability       = 0.0134

Proportion with factor
        Cases               .5
        Controls          .355     [95% Conf. Interval]
                   ---------     --------------------
        difference        .145      .0305246   .2594754
        ratio         1.408451      1.081449   1.834329
        rel. diff.    .2248062       .072871   .3767414

        odds ratio        1.58      1.094939   2.298845   (exact)

59 . mcci 25 75 43 57

                 │ Controls
Cases            │   Exposed   Unexposed  │       Total
─────────────────┼────────────────────────┼─────────────
         Exposed │        25          75  │         100
       Unexposed │        43          57  │         100
─────────────────┼────────────────────────┼─────────────
           Total │        68         132  │         200

McNemar's chi2(1) =      8.68    Prob > chi2 = 0.0032
Exact McNemar significance probability       = 0.0041

Proportion with factor
        Cases               .5
        Controls           .34     [95% Conf. Interval]
                   ---------     --------------------
        difference         .16      .0508818   .2691182
        ratio         1.470588      1.135957   1.903796
        rel. diff.    .2424242      .1020368   .3828117

        odds ratio    1.744186      1.183316   2.600014   (exact)

60 . mcci 25 75 42 58

                 │ Controls
Cases            │   Exposed   Unexposed  │       Total
─────────────────┼────────────────────────┼─────────────
         Exposed │        25          75  │         100
       Unexposed │        42          58  │         100
─────────────────┼────────────────────────┼─────────────
           Total │        67         133  │         200

McNemar's chi2(1) =      9.31    Prob > chi2 = 0.0023
Exact McNemar significance probability       = 0.0029

Proportion with factor
        Cases               .5
        Controls          .335     [95% Conf. Interval]
                   ---------     --------------------
        difference        .165      .0564947   .2735053
        ratio         1.492537      1.151972   1.933786
        rel. diff.    .2481203      .1099026    .386338
```

```
        odds ratio  1.785714      1.208114   2.671076   (exact)

61 . mcci 27 73 37 63

                 | Controls               |
Cases            |   Exposed   Unexposed  |      Total
-----------------+------------------------+-----------
         Exposed |        27          73  |        100
       Unexposed |        37          63  |        100
-----------------+------------------------+-----------
           Total |        64         136  |        200

McNemar's chi2(1) =      11.78    Prob > chi2 = 0.0006
Exact McNemar significance probability       = 0.0008

Proportion with factor
        Cases               .5
        Controls           .32     [95% Conf. Interval]
                     ---------     --------------------
        difference         .18      .0752919   .2847081
        ratio           1.5625      1.208444   2.020289
        rel. diff.    .2647059      .1350966   .3943152

        odds ratio    1.972973      1.311021   3.014824   (exact)

62 . mcci 40 60 38 62

                 | Controls               |
Cases            |   Exposed   Unexposed  |      Total
-----------------+------------------------+-----------
         Exposed |        40          60  |        100
       Unexposed |        38          62  |        100
-----------------+------------------------+-----------
           Total |        78         122  |        200

McNemar's chi2(1) =       4.94    Prob > chi2 = 0.0263
Exact McNemar significance probability       = 0.0334

Proportion with factor
        Cases               .5
        Controls           .39     [95% Conf. Interval]
                     ---------     --------------------
        difference         .11       .009192    .210808
        ratio         1.282051      1.029187   1.597042
        rel. diff.    .1803279      .0363415   .3243142

        odds ratio    1.578947      1.034531   2.436768   (exact)

63 . mcci 32 57 68 43

                 | Controls               |
Cases            |   Exposed   Unexposed  |      Total
-----------------+------------------------+-----------
         Exposed |        32          57  |         89
       Unexposed |        68          43  |        111
-----------------+------------------------+-----------
           Total |       100         100  |        200

McNemar's chi2(1) =       0.97    Prob > chi2 = 0.3252
Exact McNemar significance probability       = 0.3712
```

Proportion with factor

Cases	.445		
Controls	.5	[95% Conf.	Interval]
difference	-.055	-.1692998	.0592998
ratio	.89	.7055259	1.122709
rel. diff.	-.11	-.3408684	.1208684
odds ratio	.8382353	.5789865	1.209703 (exact)

64 . mcci 32 68 57 43

Cases	Controls Exposed	Unexposed	Total
Exposed	32	68	100
Unexposed	57	43	100
Total	89	111	200

McNemar's chi2(1) = 0.97   Prob > chi2 = 0.3252
Exact McNemar significance probability = 0.3712

Proportion with factor

Cases	.5		
Controls	.445	[95% Conf.	Interval]
difference	.055	-.0592998	.1692998
ratio	1.123596	.890703	1.417382
rel. diff.	.0990991	-.0882789	.2864771
odds ratio	1.192982	.8266492	1.727156 (exact)

65 .
