# Supplementary material for: Effect of Healthy Transitions intervention in improving family planning uptake among adolescents and young women in Western Nepal: A pre-and post-intervention study
Source: PLoS One. 2023 Jun 9;18(6):e0286705. doi: 10.1371/journal.pone.0286705 (PMC10256217; doi:10.1371/journal.pone.0286705)
Supplement: Attachment — Submitted filename: Response to Reviewers.docx [file pone.0286705.s005.docx]

Feb 14, 2023

Dear Editors-in-Chief

PLOS ONE

Thank you very much for your email with the decision dated Jan 24, 2023. We found that reviewers' and editor feedbacks were insightful. We have addressed reviewers’ comments point by point. We have uploaded the revised manuscript showing track changes so that you can see all revisions and modifications we have made. We believe that our revisions will satisfy you and both reviewers. We have included a clean copy and track change copy of the revised manuscript.

Thank you for considering this manuscript for publication.

Sincerely,

Dipendra Singh Thakuri

On behalf of all co-authors

**Reviewer #2:**

The title should be modified as follows: “Implementation of the Health Transition Project has improved family planning uptake among adolescents and young women in Western Nepal: a pre-and post-intervention study.”

***Authors response****: Thank you so much for important suggestions/inputs. We have modified the title incorporating your suggestions. The modified title is “Effect of Healthy Transitions intervention in improving family planning uptake among adolescents and young women in Western Nepal: a pre-and post-intervention study”.*

What happen to the rest of the participants who were involved at the beginning of the intervention. From 786-565 girls, why?

***Authors response****: Thank you for your insightful comment. Rest of the participants (221 AGYW) who involved in the baseline were lost to follow up. The losses observed between baseline and endline were due to adolescent girls pursuing higher secondary education, some married adolescent girls moved to India with their husbands for seasonal labor work and remaining were not surveyed because of their inconsistent participation in the project. And these losses were random.*

Where is the figure??

***Authors response****: Thank you for your query. As per the journal guideline, the figure supposed to be uploaded as separate file, so it is uploaded as separate file and not included in the main manuscript.*

No abbreviation in subtitles or titles.

***Authors response****: Thank you for important suggestion. We have removed abbreviations from subtitle and titles as suggested.*

Also decode them when you use them the first time.

***Authors response****: Thank you for your suggestions. We have revised it as suggested.*

Not clear or needs some modification.

***Authors response****: Thank you so much for your important comment. We have rephrased it as suggested.*

One and half page would be enough

***Authors response****: Thank you so much for your feedback.*

??? Difference

***Authors response****: Thank you for* your query. The difference is from baseline to endline.

Why did you have to wait 12 months? Six months would be enough, or it would be nice to see the sixth month record as well as the twelfth month to also show how the trend goes?

***Authors response****: Thank you for your important inquiry. The Healthy Transition curriculum consisted of a total of 24 sessions, which were conducted among AGYW on a fortnightly basis, so it took one year to cover the entire package of Healthy Transition, so we had to wait 12 months to complete the full intervention package.*

The similarities in the proportions at each category at the baseline and endline survey brings a question of trustworthiness of the data.

***Authors response****: Thank you so much for comment. We followed the same groups of participants from baseline to endline, and a similar proportion of each category may have been lost, so there may not have been many differences between baseline and endline in each category.*

We would like to thank the editor and both the reviewers for their insightful comments and feedback. Thank you so much for inviting us to revision of this manuscript.

Dipendra Singh Thakuri, on behalf of all co-authors
