# Supplementary material for: Effect of Healthy Transitions intervention in improving family planning uptake among adolescents and young women in Western Nepal: A pre-and post-intervention study
Source: PLoS One. 2023 Jun 9;18(6):e0286705. doi: 10.1371/journal.pone.0286705 (PMC10256217; doi:10.1371/journal.pone.0286705)
Supplement: Attachment — Submitted filename: Response to Reviewers.docx [file pone.0286705.s006.docx]

April 3, 2023

Dear Editors-in-Chief

PLOS ONE

Thank you very much for your email with the decision dated March 21, 2023. We found that editor feedbacks were insightful. We have addressed editor comments point by point. We have uploaded the revised manuscript showing track changes so that you can see all revisions and modifications we have made. We believe that our revisions will satisfy you. We have included a clean copy and track change copy of the revised manuscript.

Thank you for considering this manuscript for publication.

Sincerely,

Dipendra Singh Thakuri

On behalf of all co-authors

**Editor comments:**

We advise that authors should get an experienced biostatistician to review the data analysis especially Tables 3 and Table 4. For comparison of categorical variable of baseline and endline, a Pearson's Chi-square may not be appropriate because there is a violation of the assumption of "independent" groups. A McNamar test appears to be more appropriate while paired t-test is more appropriate for a continuous variable that is normally distributed.

***Author’s response:*** Thank you so much for your insightful comment. We have consulted with a biostatistician to review the data analysis. Based on the suggestions, we did a re-analysis of Table 3 and Table 4 using the McNamar test and reported the findings in the revised manuscript.

In Table 3 frequencies were stated without percentages.  In the column for "Diff", some places had frequencies. While others had percentages. It makes the Table quite difficult to understand. Authors should revise this.

***Author’s response****: Thank you so much for your suggestions. We have reviewed and revised it as per the suggestions.*

The calculation of absolute difference and corresponding P-value was not stated in the statistical analysis section. Authors should explain fully what statistical analysis that was done (The statistical steps should be apparent/ more explicit). I reckon that the P-value obtained from a Chi-square does not apply to absolute difference of categorical values and/or frequencies as depicted and interpreted in the result. Authors should get a biostatistician to review the data analysis and result presentation so that valid conclusion can be obtained from the data/result.

***Author’s response:*** Thank you for your important comments. We have added details about statistical analysis in the statistical analysis section of the revised manuscript. In terms of data analysis, we consulted with the biostatistician and reran the analysis of the data using appropriate statistical test.

Table 4. Title. Write "FP" in full.

Table 4 also requires overhaul of the statistical analysis.

**Author’s response:** *Thank you for your suggestions. We have reviewed and revised it as per your suggestions.*

We would like to thank the editor and both the reviewers for their insightful comments and feedback. Thank you so much for inviting us to revision of this manuscript.

Dipendra Singh Thakuri, on behalf of all co-authors
